# Family-Four Aldehyde Dehydrogenases Play an Indispensable Role in the Pathogenesis of *Magnaporthe oryzae*

**DOI:** 10.3389/fpls.2018.00980

**Published:** 2018-08-08

**Authors:** Waheed Abdul, Sami R. Aliyu, Lili Lin, Malota Sekete, Xiaomin Chen, Frankline J. Otieno, Tao Yang, Yahong Lin, Justice Norvienyeku, Zonghua Wang

**Affiliations:** ^1^Fujian University Key Laboratory for Functional Genomics of Plant Fungal Pathogens, College of Life Sciences, Fujian Agriculture and Forestry University, Fuzhou, China; ^2^Fujian and Taiwan Joint Center for Ecological Control of Crop Pests, College of Plant Protection, Fujian Agriculture and Forestry University, Fuzhou, China; ^3^Department of Crop Science, Faculty of Agriculture, National University of Lesotho, Lesotho, Southern Africa; ^4^Institute of Oceanography, Minjiang University, Fuzhou, China

**Keywords:** aldehyde dehydrogenase, free fatty acid radicals, lipid peroxidation, aldehydes, *Magnaporthe oryzae*

## Abstract

The oxidative degradation of lipids through lipid peroxidation processes results in the generation of free fatty acid radicals. These free radicals including reactive oxygen species (ROS) serve as a substrate for generating reactive aldehydes. The accumulation of free fatty acid radicals, ROS, and reactive aldehydes in cell compartments beyond physiological threshold levels tends to exert a damaging effect on proximal membranes and distal tissues. Living organisms deploy a wide array of efficient enzymes including superoxide dismutase (*SOD*), catalase (*CAT*), peroxidase (*POD*), and aldehyde dehydrogenases (*ALDHs*) for scavenging reactive molecules and intermediates produced from membrane lipid peroxidation events. Although the contributions of *SOD*, *CAT*, and *POD* to the pathogenesis of microbial plant pathogens are well known, the influence of *ALDH* genes on the morphological and infectious development of plant pathogenic microbes is not well understood. In this study, we deployed RNA interference (RNAi) techniques and successfully silenced two putative family-four aldehyde dehydrogenase genes potassium-activated aldehyde dehydrogenase (*MoKDCDH*) and delta-1-pyrrorine-5-carboxylate dehydrogenase (*MoP5CDH*) in the rice blast pathogen *Magnaporthe oryzae*. The results obtained from the phenotypic analysis of individual knock-down strains showed that the RNAi-mediated inactivation of *MoKDCDH* and *MoP5CDH* triggered a significant reduction in conidiogenesis and vegetative growth of Δ*Mokdcdh* and Δ*Mop5cdh* strains. We further observed that downregulating the expression of *MoKDCDH* and *MoP5CDH* severely compromised the pathogenesis of the rice blast fungus. Also, the disruption of *MoKDCDH* and *MoP5CDH M. oryzae* undermined membrane integrity and rendered the mutant strains highly sensitive to membrane stress inducing osmolytes. However, the *MoKDCDH* and *MoP5CDH* knock-down strains generated in this study displayed unaltered cell wall integrity and thus suggested that family-four *ALDH*s play a dispensable role in enforcing cell wall-directed stress tolerance in *M. oryzae*. From these results, we deduced that family-four *ALDH*s play a conserved role in fostering membrane integrity in *M. oryzae* possibly by scavenging reactive aldehydes, fatty acid radicals, and other alcohol derivatives. The observation that downregulating the expression activities of *MoKDCDH* had a lethal effect on potential mutants further emphasized the need for comprehensive and holistic evaluation of the numerous *ALDH*s amassed by the rice blast fungus for their possible engagement as suitable targets as antiblast agents.

## Introduction

Aldehydes are intermediates in several fundamental metabolic pathways, including the synthesis of carbohydrates, vitamins, steroids, amino acids, and lipids (Kirch et al., 2004). Aldehydes also accumulate in tissues in response to environmental stresses, including salinity, dehydration, desiccation, cold, and heat shock (Feder and Hofmann, 1999). Aldehyde molecules are reactive at excessive physiological concentrations and hence tend to negatively impact on cell growth, yield, seed survival, and membrane integrity (Kotchoni et al., 2010). Living organisms tightly regulate the cellular aldehyde level by limiting membrane peroxidation events by exploring antioxidant properties of either superoxide dismutase (*SOD*), catalase (*CAT*), or peroxidase (*POD*) (Schnitzer et al., 2007; Hossain et al., 2015). Furthermore, the direct regulation of cellular aldehyde levels involves enzymatic activities of aldehyde dehydrogenases (Singh et al., 2013).

Aldehyde dehydrogenases (*ALDH*s) are a group of evolutionarily conserved polymorphic enzymes (Zhu et al., 2014). *ALDH*s belong to a superfamily of NADP^+^-dependent enzymes that are involved in the irreversible oxidation of endogenous and exogenous aldehydes to their corresponding carboxylic acids (Zhang et al., 2012; Luo et al., 2015; Buchman and Hurley, 2017). A total of 19 dehydrogenase-encoding genes have been recorded in the human genome. Insights gained from these previous studies showed that human *ALDH*s play critical cellular and biological roles including detoxification of aldehydes, cell proliferation, peroxidation of membrane lipid, protection of tissues against hyperosmotic pressure, and inhibition of tumors and cancers (Januchowski et al., 2013; Morgan and Hurley, 2015).

The research evidence currently available also showed that *ALDH*s promote abiotic stress tolerance, facilitate the restoration of male sterility, and regulate embryo development and seed viability as well as maturation in different plant species including rice (Kotchoni et al., 2010; Gill, 2014). Although plants have several distinct types of *ALDH*s, information regarding their individual and collective biological role is limited and requires further investigations (Kotchoni et al., 2006; Stiti et al., 2011).

Previous studies have also shown that reactive oxygen species (ROS) and other reactive molecules including aldehydes play dual functions during host–pathogen interaction. Plants accumulate free fatty acid radicals and other derivatives of membrane lipid peroxidation processes as a significant and earliest pathogen-triggered immune (PTI) response (Palukaitis and Carr, 2008; Heller and Tudzynski, 2011; Ranf et al., 2011; Smith and Heese, 2014; Lehmann et al., 2015). Under this situation, plants generate free radicals and reactive aldehydes as direct reactive substrates to kill pathogens or as secondary defence signaling molecules for activating durable host resistance against invading pathogens (Norvienyeku et al., 2017). Pathogens, in contrast, generate and secrete a wide range of free fatty acid radicals, ROS, reactive aldehydes, and alcohol derivatives that are functionally and structurally similar to reactive molecules produced by plants as virulence factors during host–pathogen interactions (Heller and Tudzynski, 2011; Huang et al., 2011). The majority of these reactive molecules have a damaging effect on cell wall and cell membranes (Thannickal and Fanburg, 2000). During host-pathogen interaction, microbial pathogens secretes reactive molecules into host cells to successfully suppress host immunity (Koop, 2006; Bryan et al., 2012).

Many research findings have shown that plants, as well as microbial pathogens, deploy *SOD*, *CAT*, and *POD* for ROS scavenging. Although aldehydes are known as one of the most diverse lipid oxidation products (LOPs), the knowledge on aldehyde scavenging activities of aldehyde dehydrogenases during growth, reproduction, and infectious development is not extensively studied in filamentous phyto-pathogenic fungi.

In our previous study, we showed that the filamentous ascomycete fungus *Magnaporthe oryzae* possesses a total of 16 *ALDH*s in its genome (Norvienyeku et al., 2017). *ALDH* genes are prone to gene duplication events (Zhang et al., 2012). Moreover, records from our previous studies showed that the 12 of the 16 *ALDH* genes identified in *M. oryzae* experienced gene duplication while the remaining 4 genes, including methylmalonate-semialdehyde dehydrogenase (*MoMSDH/MoMMSDH*), betaine aldehyde dehydrogenase1 (*MoBADH1*), potassium-activated aldehyde dehydrogenase (*MoKDCDH*), and delta-1-pyrrorine-5-carboxylate dehydrogenase (*MoP5CDH*), exist as single copies. We also showed that the targeted deletion of *MoMSDH* severely compromised sporulation, germination, appressorium morphogenesis, redox homeostasis, and pathogenesis of Δ*Momsdh* strain (Norvienyeku et al., 2017).

The additional research also showed that the disruption of succinate semialdehyde dehydrogenase 2 (*MoSSADH2*) in the rice blast fungus equally triggered a significant reduction in growth and completely abolished sporulation in defective strains; all these studies showed that *ALDH*s might assume roles beyond detoxification of endogenously generated and exogenously trans-located reactive aldehydes.

In this study, we silenced the remaining single copy of *ALDH* genes (*MoKDCDH*, *MoP5CDH*, and *MoBADH1*) using RNA interference (RNAi) strategy. We proceeded further to evaluate growth, sporulation, and infection characteristics of the Δ*Mokdcdh* and Δ*Mop5cdh* strains. The corresponding results obtained from this investigation showed that the silencing of *MoKDCDH* and *MoP5CDH* exerts adverse effects on growth, sporulation, and pathogenesis of *MoKDCDH* and *MoP5CDH* knock-down strains. However, for some unknown reason, attempts aimed at silencing *MoBADH1* were unsuccessful.

## Results and Discussion

### Domain Homology and Architecture of *M. oryzae* Aldehyde Dehydrogenases

Aldehyde dehydrogenases are evolutionarily conserved and ubiquitously present in all living organisms across kingdoms. Most members of the *ALDH* superfamily are associated with high incidences of gene duplication (Brocker et al., 2013). In our previous investigation, we identified a total of 16 *ALDH* (*MoALDH*)-encoding genes in the rice blast fungus genome and further demonstrated that 12 of the 16 *ALDH*s identified in *M. oryzae* have multiple copies (Norvienyeku et al., 2017).

**FIGURE 1:**
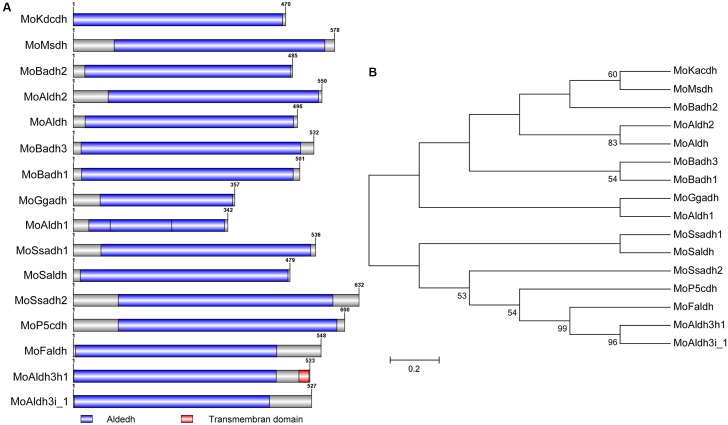
Domain architecture and domain base phylogeny of *MoALDHs*. **(A)** Structure dynamics of the conserved Aldedh-domain predicted for the 16 *ALDHs* identified in *M. oryzae* genome. **(B)**. Domain sequence-mediated clustering and maximum-likelihood phylogeny of all the 16 *M. oryzae* aldehyde dehydrogenases tested with 1,000 bootstrap replicates.

To provide insights into inherent cladistic relationship prevailing between the respective *MoALDH*s, we performed Pfam^1^ and SMART^2^-based assisted domain prediction analysis for all the 16 *M. oryzae ALDH*s. The results obtained from domain prediction analysis showed that all the 16 *MoALDHs* possessed the conserved Aldedh domain (**Figure 1A**), and given this observation, we accordingly inferred that the Aldedh domain likely represents a basic but indispensable genetic parameter that defines aldehyde-catalyzing capabilities of the respective Aldedhs. We additionally performed phylogenetic and domain homology analyses using amino acid (aa) sequences of the Aldedh-domain region of all the 16 *M. oryzae ALDHs*. The phylogenetic cladogram obtained from this examination revealed that based on the domain sequence homology, *MoALDHs* could be grouped into nine distinct and diverse subfamilies (**Figure 1B**). The high and diverse number of clusters recorded from the bootstrap maximum-likelihood analysis conducted in this study could be attributed to the fact that the traditional classification of *ALDHs* was based on their requirement for NAD/NADP^+^ as a reaction co-factor rather than conserved Aldedh domain (Perozich et al., 1999). Further, the subfamily-level mapping was carried out by conducting homologous and orthologous BLASTP search with domain sequences of respective *MoALDHs* in the human genome database^3^ and *ALDH*^4^ website, Arabidopsis genome database^5^, and rice genome database^6^. The subfamily level classification results showed that the 16 *MoALDH* genes belong to eight different *ALDH* subfamilies (**Table 1**). Since different members of the *ALDH* family acts on different types of aldehydes as substrates (Jimenez-Lopez et al., 2016), we subsequently posited that phylogeny, domain structure, and wide subfamily-level divergence exhibited by the various *MoALDH* genes are likely a reflection of their functional divergence.

**Table 1:** Family level classification of *M. oryzae* aldehyde dehydrogenase (*MoALDHs*).

Family	Accession No.	Actual Name	Given Name
Family 1	*Mgg_03900*	Aldehyde dehydrogenase	ALDH
Family 2	*Mgg_13331*	Gamma-glutamyl-gamma-aminobutyraldehyde dehydrogenase	GGADH
	*Mgg_06551*	Aldehyde dehydrogenase	ALDH1
Family 3	*Mgg_07270*	Fatty aldehyde dehydrogenase	FALDH
	*Mgg_00719*	Aldehyde dehydrogenase 3H1	ALDH3H1
	*Mgg_07890*	Aldehyde dehydrogenase	ALDH3I_1
Family 4	*Mgg_05814*	Potassium-activated aldehyde dehydrogenase	KACDH
	*Mgg_17513*	Delta-1-pyrrorine-5-carboxylate dehydrogenase	P5CDH
Family 5	*Mgg_00652*	Salicylaldehyde dehydrogenase	SALDH
	*Mgg_01230*	Succinate-semi aldehyde dehydrogenase	SSADH1
	*Mgg_02766*	Succinate-semi aldehyde dehydrogenase	SSADH2
	*Mgg_05008*	Aldehyde dehydrogenase	ALDH2
Family 6	*Mgg_01606*	Methylmalonate-semialdehyde dehydrogenase	MSDH
Family 9	*Mgg_09456*	Betaine aldehyde dehydrogenase	BADH1
Family 10	*Mgg_03263*	Betaine aldehyde dehydrogenase	BADH2
	*Mgg_01991*	Betaine aldehyde dehydrogenase	BADH3

### Generation of *MoKDCDH* and *MoP5CDH* Knock-Down Mutants

**FIGURE 2:**
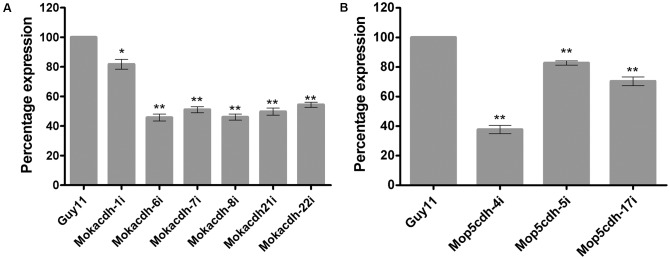
Percentage (%) fold expression of *MoKDCDH* and *MoP5CDH* in respective knock-down mutants. **(A)** The expression level of *MoKDCDH* in six different independent knock-down mutants of *MoKDCDH*. **(B)** The fold expression of *MoP5CDH* in the three independent *MoP5CDH* knock-down mutants. The quantitative real-time PCR (qRT-PCR) data were computed with Microsoft Excel spread sheet in conjuction with graphpad prism6. The error bars represent mean ± SD, whereas single and double asterisks “^∗^” represent a significant reduction in the fold expression of *MoKDCDH* and *MoP5CDH* in their respective knock-down strains. Consistent values were obtained with five independent biological replications and three technical replicates for each independent qRT-PCR experiment.

In our previous studies, we showed that the expression activities of *MoKDCDH* and *MoP5CDH* fluctuated significantly during tissue invasion and colonization stages of the rice blast fungus. To assess the influence of these substrate-specific *ALDH* genes on the physiological and infectious development of the rice blast fungus, we initially deployed homologous recombination strategy in an attempt to generate *ALDH*-targeted gene deletion strains for *MoKDCDH* and *MoP5CDH*. However, we could not obtain positive gene replacement mutants for the two *ALDH* genes even after a series of repeated protoplast transformations followed by the screening of more than 2000 potential candidates for each gene. We subsequently adopted RNAi approach to generate knock-down mutants by transforming *MoKDCDH* and *MoP5CDH* knock-down plasmid vector construct into *M. oryzae* Guy11 strain. The corresponding results obtained from a qPCR-mediated screening of prospective *MoKDCDH* and *MoP5CDH* gene knock-down transformants revealed six (6) and three (3) independent knock-down mutants for *MoKDCDH* and *MoP5CDH*, respectively (**Figures 2A,B**). Also to assess the influence (off-target effect) of RNAi-mediated disruption of *MoKDCDH* and *MoP5CDH* on other members of the *ALDH* family, we monitored the transcriptional activities of individual members of *M. oryzae ALDH* superfamily in each independent knock-down strain relative to the wild-type strain using qPCR. Results obtained from these analyses revealed that the disruption of *MoKDCDH* and *MoP5CDH* did not significantly alter the expression pattern of other members of the *ALDH* superfamily (Supplementary Figure S1) indicating that the silencing of *MoKDCDH* and *MoP5CDH* had no off-target effect on other *ALDH*s in *M. oryzae.*

Our studies revealed that *MoKDCDH* shared the same subfamily (family-four) with *MoP5CDH*, and records have shown that the deletion of *MoP5CDH* ortholog *DmP5CDh1* in *Drosophila melanogaster* triggered proline accumulation and swollen mitochondria and subsequently resulted in larval and pupal lethality (Koštál et al., 2012). This knowledge coupled with our inability to generate *MoKDCDH* and *MoP5CDH*-targeted gene replacement mutants subsequently informed our speculative conclusion that *MoKDCDH* and *MoP5CDH* likely play indispensable roles in the survival of the rice blast fungus.

### Family-Four *ALDHs* Are Required for the Survival, Growth, Sporulation, and Pathogenesis of *M. oryzae*

**FIGURE 3:**
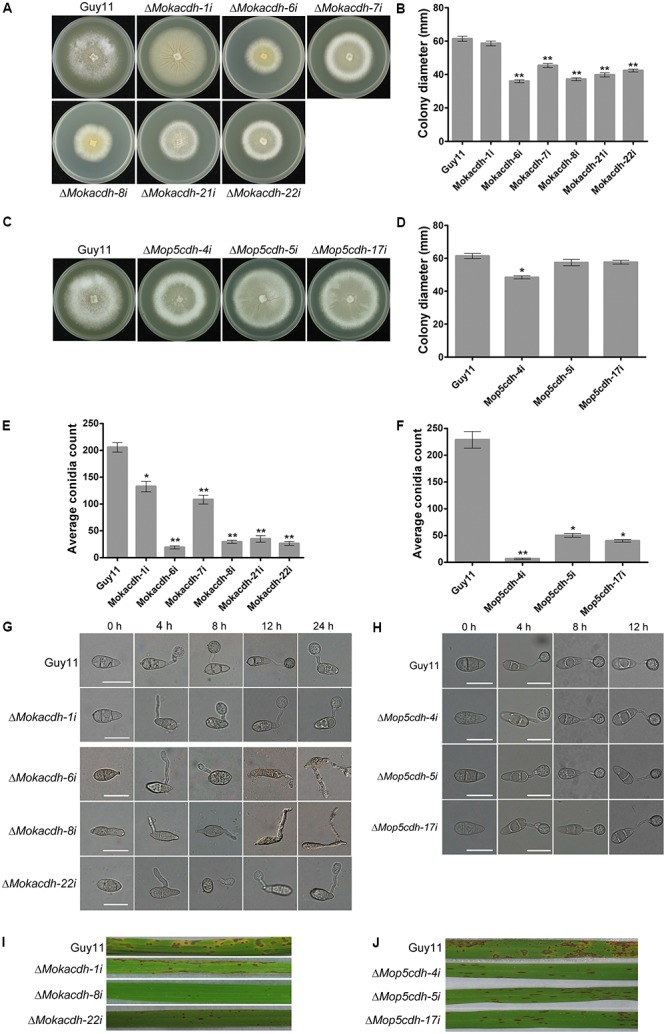
Family-four aldehyde dehydrogenases play indispensable role in survival, growth, sporulation, and pathogenesis of *M. oryzae*. **(A)** Average colony diameters of Δ*Mokdcdh* knock-down strains and the wild-type strain cultured on CM media for 10 days. **(B)** Statistical evidence of growth defects exhibited by the six independent Δ*Mokdcdh* knock-down strains compared with the wild-type strain. **(C)** The average colony diameters of *MoP5CDH* knock-down strains and the (wild-type strain cultured on CM media for 10 days. **(D)** The statistical evaluation of the vegetative growth level recorded in the Δ*Mop5cdh* knock-down strains compared with the wild-type strain. **(E)** Conidiation abilities of the respective Δ*Mokdcdh* knock-down strains and the wild-type strain cultured under the same growth conditions. **(F)** Statistical presentation of conidiation characteristics of the various Δ*Mop5cdh* knock-down strains compared with the wild-type strains cultured under similar growth conditions. **(G)** The different types of conidia abnormalities as well as germination and appressorium abnormalities exhibited by the six independent Δ*Mokdcdh* knock-down strains. **(H)** Morphology conidia, conidia germination, and appressorium formation characteristics exhibited by the three independent *MoP5CDH* knock-down mutants generated in this study. **(I)** Results obtained from infection assays conducted by spray inoculation susceptible CO39 rice seedlings with conidia obtained from the six independent Δ*Mokdcdh* knock-down strains along with the wild-type conidia as control. **(J)** Infection capabilities of the three *MoP5CDH* knock-down strains. Both statistical and nonstatistical experimental data were generated from three independent biological experiments with three replicates each time with consistent results. One-way ANOVA (nonparametric) statistical analysis was carried out with graphpad prism6 and Microsoft Excel spreadsheet, and the error bars represent the standard deviation. Single and double asterisk(s) “^∗^” represent significant differences existing between Guy11 and the respective knock-down mutants (*P* < 0.05) and (*P* < 0.01).)

Upon successful silencing of *MoKDCDH* and *MoP5CDH*, we assayed the contribution of *MoKDCDH* and *MoP5CDH* to the vegetative growth and morphological development of *M. oryzae* by culturing *MoKDCDH* and *MoP5CDH* knock-down mutants and the wild-type strain on complete media (CM) for 10 days under 28°C before proceeding to measure the colony diameter of respective mutants. The results obtained from these examinations showed that downregulating the activities of *MoKDCDH* and *MoP5CDH* resulted in a corresponding reduction in the vegetative growth of the individual gene knock-down mutants (**Figures 3A–D**). The seeming correlation observed between the expression levels of *MoKDCDH* and *MoP5CDH*, their respective knock-down lines, and the magnitude of growth reduction informed our conclusion that the silencing of *MoKDCDH* and *MoP5CDH* triggered the growth defects exhibited by the Δ*Mokdcdh* and Δ*Mop5cdh* strains.

To further unravel the influence of *MoKDCDH* and *MoP5CDH* on the generation of asexual spores in the rice blast fungi, we instituted relevant sporulation assays to examine the quantum and morphology of asexual spores generated by *MoKDCDH* and *MoP5CDH* knock-down mutants relative to the wild type. The results obtained from these investigations showed that the RNAi-mediated silencing of *MoKDCDH* and *MoP5CDH* drastically compromised sporulation characteristics of both *MoKDCDH* and *MoP5CDH* knock-down mutants (**Figures 3E,F**). We also showed that *MoKDCDH* exclusively played a role in modulating conidia morphogenesis; hence, *MoKDCDH* knock-down mutants displayed abnormal conidium morphogenesis, conidium septation, germination, and appressorium morphogenesis (**Figure 3G**). Conversely, *MoP5CDH* knock-down strains displayed intact conidia and appressorium morphology with no visible septation and germination defects (**Figure 3H**), and we further observed that downregulating the expression activities of *MoKDCDH* to levels below 50% had lethal effect. Hence, the resultant mutants displayed short lifespan (could not be stored beyond 3 months). From these results, we inferred that *MoKDCDH* likely mediates conidia and appressoria morphogenesis in the rice blast fungus through direct or indirect regulation independent pathways that are not under the collective influence of the family-four *ALDH*s. The additional results derived conidia-mediated infection assays showed that both *MoKDCDH* and *MoP5CDH* knock-down strains with substantially reduced expression activities failed to induce blast infection, and as a result, susceptible rice seedlings sprayed inoculated independently with conidia from *MoKDCDH* and *MoP5CDH* knock-down strains in suspension displayed reduced lesions (**Figures 3I,J**).

*MoKDCDH* and *MoP5CDH* are family-four aldehyde dehydrogenases, and we showed that *MoKDCDH* and *MoP5CDH* knock-down mutants with <50% expression activity were nonpathogenic. Growth records obtained from this study showed that the silencing of *MoKDCDH* and *MoP5CDH* also caused a significant reduction in vegetative growth and adequately support our conclusion that the full participation of family-four aldehyde dehydrogenases (potassium-activated aldehyde dehydrogenase and delta-1-pyrrorine-5-carboxylate dehydrogenase) is likely necessary for growth, virulence, and pathogenicity of the rice blast fungus during host–pathogen interaction. This position is firmly supported by previous research findings, which equally showed that potassium-activated aldehyde dehydrogenase in *Colletotrichum acutatum* functions as a pathogenicity factor that promoted disease development (Guidarelli et al., 2011).

### Response of *MoKdcdh* and *MoP5Cdh* Knock-Down Mutants to Oxidative Stress

Since aldehyde dehydrogenases are inherently involved in the peroxidation of membrane lipids coupled with the fact that the peroxidation of membrane lipid enhances membrane integrity and bolsters cell membrane against oxidative stress.

We subsequently decided to assess the response of *MoKDCDH* and *MoP5CDH* knock-down mutants by culturing respective knock-down lines along with the wild-type strain on CM supplemented with oxidative and reductive stress-inducing agents including acetaldehydes, alcohol, SDS, ROS, and DTT. The individual knock-down mutants were highly sensitive to SDS, ROS, and NaCl (oxidative stress) (Trotter and Grant, 2002; Vaidyanathan et al., 2003; Furukawa et al., 2017; Conrad et al., 2018) and *MoP5CDH* and DTT (reductive stress) (Trotter and Grant, 2002; Valadi et al., 2004). From these results, we concluded that *MoKDCDH* and *MoP5CDH* promote oxidative and reductive stress tolerance of *M. oryzae.*

**FIGURE 4:**
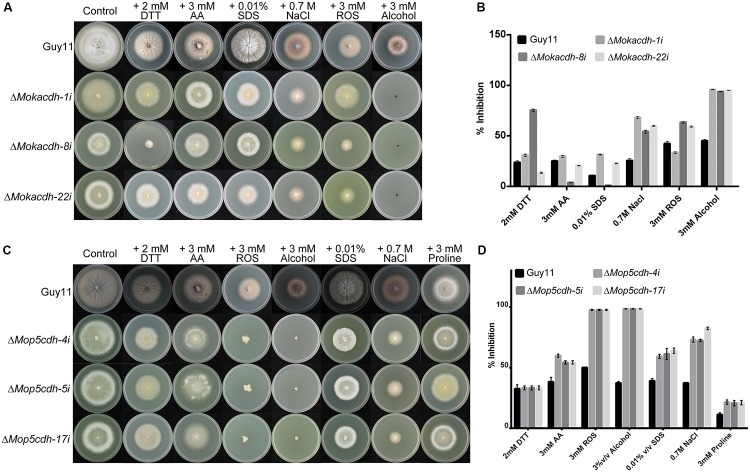
Family-four aldehyde dehydrogenases contribute oxidative and reductive stress tolerance in *M. oryzae*. **(A)** Growth response of Δ*Mokdcdh* knock-down strains and the wild-type strain on CM supplemented with 3 mM H_2_O_2_, 3% ^v^/_v_ alcohol, 0.7 M NaCl, 0.01% ^v^/_v_ SDS, 2 mM DTT, and 3 mM acetaldehyde, as osmolytes with the aim to assess the contribution of *MoKDCDH* to stress tolerance of *M. oryzae*. **(B)** The percent inhibition of Δ*Mokdcdh* knock-down strains and the wild-type strain on CM supplemented with 3 mM H_2_O_2_, 3% ^v^/_v_ alcohol, 0.7 M NaCl, 0.01% ^v^/_v_ SDS, 2 mM DTT, and 3 mM acetaldehyde. **(C)** Sensitivity of Δ*Mop5cdh* knock-down strains along with the wild-type strain to 3 mM H_2_O_2_, 3% ^v^/_v_ alcohol, 0.7 M NaCl, 0.01% ^v^/_v_ SDS, 2 mM DTT, and 3 mM acetaldehyde. **(D)** The percent inhibition of respective Δ*Mop5cdh* knock-down strains and the wild-type strain grown on CM supplemented with 3 mM H_2_O_2_, 3% ^v^/_v_ alcohol, 0.7 M NaCl, 0.01% ^v^/_v_ SDS, 2 mM DTT, and 3 mM acetaldehyde. One-way ANOVA (nonparametric) was used in the statistical evaluation of percent inhibition, computational analysis was performed with graphpad prism6 software and Microsoft Excel spreadsheet, and the error bars represent the standard deviation. The percent inhibition was obtained by the formula: Inhibition rate = (the diameter of untreated strain – the diameter of treated strain)/(the diameter of untreated strain × 100%)].

Furthermore, *MoKDCDH* and *MoP5CDH* knock-down strains displayed higher sensitivity toward alcohol and ROS (**Figures 4A–D**). Records currently available showed that *P5CDH* enforces membrane integrity and oxidative stress tolerance by catalyzing the transformation of delta-1-pyrroline-5-carboxylate derived from proline or ornithine into glutamate (Hirayama-Kurogi et al., 2017). Therefore, we subsequently posited that the two family-four aldehyde dehydrogenases retain and play their conserved role in the detoxification of ROS, reactive aldehydes, and other alcohol derivatives generated during the morphological, physiological, and infectious development of *M. oryzae.*

## Conclusion

Aldehyde dehydrogenases (*ALDH*s) are an evolutionarily conserved group of NAD/NADP-dependent multigenic enzymes that are involved in the irreversible oxidation of endogenous and exogenous reactive aldehydes to their nontoxic corresponding carboxylic acids (Dong et al., 2017; Li et al., 2017; Norvienyeku et al., 2017). Members of the *ALDH* superfamily are grouped into subfamilies based on the type of conserved amino acid residue clusters found around the enzyme-binding sites (Guo et al., 2017; Jia et al., 2017; Muñoz-Clares et al., 2017; Missihoun et al., 2018). Currently, more than 25 *ALDH* subfamilies have been identified in different organisms, plant *ALDH*s have been characterized into the subfamily level in different plant species including *Solanum lycopersicum*, *Arabidopsis thaliana*, *C. reinhardtii*, *O. tauri*, the moss *P. patens*, and *Zea mays* (Kirch et al., 2004; Wood and Duff, 2009; Kotchoni et al., 2010). In humans, a total of 19 *ALDH*s have been identified and classified into 11 families and well characterized (Vasiliou et al., 2004; Zhang et al., 2012). However, fungi *ALDH*s have not received the needed attention with regard to classification and characterization. In this study, the 16 *MoALDH*s previously identified in *M. oryzae* were successfully classified into seven families. In contrast to humans, we showed that *M. oryzae* family-four *ALDH*s consists of two members, *MoP5CDH*, an ortholog of human delta-1-pyrroline-5-carboxylate dehydrogenase, and *ALDH4A1* in addition to potassium-activated aldehyde dehydrogenase (He and DiMario, 2011; Yao et al., 2013). In humans, family-four *ALDH* inactivity has been associated with seizures, mental retardation, and cellular stress tolerance (Pérez-Arellano et al., 2010). It has also been shown that the deletion of family *ALDH* in *Drosophila melanogaster* triggered larval and pupal lethality (Wan et al., 2015).

Our studies showed that the successful RNAi-mediated silencing of family-four *MoALDH*s severely compromised growth, sporulation, stress tolerance, and pathogenicity of *MoKDCDH* and *MoP5CDH* knock-down mutants and subsequently confirmed previous research submissions that *P5CDH* promoted sporulation and virulence of fungal pathogens (Yao et al., 2013). These findings further showed that a complete evaluation of whole-genome aldehyde dehydrogenases in the model plant pathogenic fungi would tremendously contribute to enhance our knowledge with regard to the fundamental role of *ALDH*s during the host–pathogen interaction and their deployment as potential targets for fungicide development.

## Materials and Methods

### Strains

*Magnaporthe oryzae* wild-type strain Guy11 used in this study to generate mutant *ALDH* deletion mutants was kindly provided by Dr. Didier Tharreau (CIRAD, Montpellier, France). Competent cells used in this research for the propagation of the constructed plasmids were prepared from *Escherichia coli* (*E. coli*) strain DH5α.

### Growth Media

The Guy11 strain used in this study to generate *ALDH* mutants were cultured under an optimum temperature of 28°C on complete medium (CM: 0.6% yeast extract, 0.6% casein hydrolysate, 1% sucrose, and 1.5% agar). The *E. coli* strain DH5α used in this study to generate competent cells was cultured on lysogeny broth (LB) medium prepared by dissolving 10 g tryptone, 5 g yeast extract, and 10 g NaCl in 800 ml dH_2_O. The pH adjusted to 7 by adding 1 M NaOH in a dropwise manner before adding up more deionized water to make up to the desired volume of 1 L with dH_2_O. Both CM and LB growth media were sterilized by autoclaving at 121°C for 20 min.

### The Chemicals

All reagents and chemicals, as well as kits used in this study, are certified analytical grade, which are purchased from certified suppliers including Sigma–Aldrich, Amresco, Chromotek, and Roche.

### Primers and Plasmids

Nucleotide sequences for the individual *ALDH*s were acquired from comparative *M. oryzae* genome database established by Broad Institute^7^. The primers used for amplifying respective sequences to generate RNAi knock-down mutant strains were designed with the BACON primer designer. BGI, Shenzhen, sequenced all designed primers; a pTE11 plasmid containing neuromycine as the selective marker was used as a fusion vector for generating RNAi silence mutant strains.

### Protoplast Preparation

The Guy11 isolates were cultured or grown in liquid for 4 days in a shaking incubator at a temperature of 27.5°C at 120 rpm. Media were carefully drained off leaving the colonies behind, and the cultured colonies were then macerated by grinding. The grounded tissues were re-suspended in a fresh liquid CM and incubated in a shaking incubator operating at a speed of 120 rpm overnight at a temperature of 27.5°C. Mycelium were harvested from the liquid CM by filtering and washed with sterilized double deionized water followed by 1 M sorbitol. The tissues were then dried with the filter paper and re-suspended in 40 ml of 1 M sorbitol containing 100 mg lysing enzyme from *Trichoderma harzianum* and incubated for 3 h under 30°C at 60 rpm; protoplasting was observed on an hourly basis. Generated protoplasts were filtered through one-layer sterile Mira-cloth (Calbiochem) and centrifuged at 5000 rpm for 10 min at 4°C. The protoplast in pellets was washed twice in 1 M sorbitol STC, re-suspending in STC for final protoplast count under a microscope. Protoplast concentrations were adjusted to 1×106 and shared into 2-ml sterilized Eppendorf tubes at a volume of 250–300 μl per tube. 7% DMSO was added to unused protoplast and refrigerated at -80°C for future use. Neomycin-resistant transformants were selected on media supplemented with 200 μg/ml G418 (Invitrogen) and incubated at a steady temperature of 28°C.

### Construction of Domain Architecture, Phylogenetic Analysis, and Family-Level Classification

Amino acid sequences of all the 16 *ALDHs* identified in *M. oryzae* were acquired from the versatile fungi and oomycete genomic resource portal^8^ for the domain prediction. The domain prediction performed with the Pfam domain prediction module and confirmed in the SMART^9^ database. The domain base clustering and phylogenetic analysis of all the 16 *M. oryzae* aldehyde dehydrogenases were carried out using amino acid sequences of defined Aldedh domain regions. The obtained domain sequences were aligned using Mega version 6 muscle sequence alignment tool using a complete gap deletion approach. The maximum-likelihood method was used to generate the phylogeny. Branches of the tree were tested with 1000 bootstrap replicates. The family-level classification of *MoALDH*s was performed through ortholog BLAST and reverse blasting of *M. oryzae ALDHs* (aa) sequences in the human genome database^3^ and *ALDH*^4^ website, Arabidopsis genome database^5^, and rice genome database^6^ and mapping proteins with more than 90% identity to their corresponding ortholog families.

### Generating *ALDHs* RNAi Knock-Down Mutants

*MoKDCDH* knock-down plasmid vector construct was generated by cloning 108 base pairs (bps) of unique nonhomolog sequence amplified from the *M. oryzae* strain Guy11 cDNA into a pSD1 plasmid digested with restriction enzymes *Eco*R1 and *Xba*1. For *MoP5CDH* knock-down mutants, the silencing vector was constructed by cloning 110 bp of unique nonhomologs sequence amplified from the *M. oryzae* strain Guy11 cDNA into the pSD1 plasmid digested with restriction enzymes *Eco*R1 and *Xba*1.

### Genomic DNA Isolation From *Magnaporthe oryzae* Using SDS-CTAB Method

The Guy11 isolates were cultured or grown in liquid media for 4 days in a shaking incubator at a temperature of 27.5°C at 120 rpm. Mycelia were harvested from the liquid CM by filtering, freeze dried, frozen in liquid nitrogen, and ground into fine powder with a mortar and pestle in liquid nitrogen. The grounded mycelia were re-suspended in 1 ml (100 μl) of ice-cold lysis buffer (150 mM NaCl, 50 mM EDTA, 10 mM Tris–HCl, pH 7.4, 30 μg/ml proteinase K), transferred into a 1.5-ml Eppendorf tube, and kept at 4°C to prevent endonuclease activity during rehydration of the sample. SDS was added to a final concentration of 2%, vortexed, and incubated for 30 min at 65°C in a water bath. After centrifugation for 15 min at 14,000 rpm, the supernatant was transferred to a new sterile 1.5-ml Eppendorf tube. The volume of supernatant was measured, and the NaCl with a concentration of 1.4 M and one-tenth volume of 10% CTAB buffer (10% CTAB, 500 mM Tris–HCl, 100 mM EDTA, pH 8) were added. The solution was thoroughly mixed and incubated for 10 min at 65°C in a water bath. After cooling for 2 min at 15°C, an equal volume of chloroform-isoamyl alcohol (24:1 v/v) was added, thoroughly mixed, and the tube was centrifuged for 15 min at 14,000 rpm. Extraction was repeated until the interface was clear. The supernatant was then pipetted into a new 1.5-ml Eppendorf tube, containing two volumes of cold 100% ethanol. After DNA precipitation, the pellet was centrifuged for 15 min at 4°C and 14,000 rpm. Pellets obtained after centrifugation were washed with 70% ethanol and dried at room temperature. The resultant product was re-suspended in 100 μl TE buffer with 0.002% RNAse (5 μg/ml) and incubated for 1 h at 37°C. The suspension was then used as the template for amplifying required *ALDH* fragments for intended purposes. The remaining suspension was stored at -20°C for later PCR amplification and other use.

### Extracting Total RNA From *Magnaporthe oryzae*

The wild-type Guy11 strain and *MoKDCDH* and *MoP5CDH* knock-down mutants were cultured or grown in the liquid CM for 4 days in a shaking incubator at a temperature of 27.5°C at 12,000 rpm. The cultured colonies were then filtered out, washed with sterilized double deionized water, dried, and blended in liquid nitrogen. An equal weight of the grounded samples was transferred into 1.5-ml sterilized Eppendorf tubes, suspended with 1 ml RNAiso, and vortexed vigorously to yield a uniform mixture. The mixture was then placed on the ice briefly and re-vortexed and allowed to settle for 5 min at room temperature. 200 μl of chloroform was added to the mix and vortexed for 15 s to get rid of proteins. The contents were allowed to stand still for 3 min at room temperature before proceeding to centrifugation for 15 min at 12,000 rpm under 4°C. 400 μl of the supernatant was pipetted into new sterilized Eppendorf tubes, and 400 μl isopropanol was added, gently mixed, and allowed to stand at room temperature for 10 min. The suspension was subjected to centrifugation at 12,000 rpm for 10 min at 4°C; the supernatant was discarded leaving the pellets at the bottom. Washing was done by adding 1 ml of 75% alcohol to the pellets, and centrifugation was done at 12,000 rpm for 5-min at 4°C, supernatant was discarded after centrifugation, and the precipitates were air dried under room temperature for 5 min. Dried pellets were diluted with RNAse-free water, 10X reaction buffer, and DNAse to prepare an initial solution of 200 μl in the ratio of 89:10:1 and incubated for 30 min at 37°C. After incubation, the solution was heated to a temperature of 65°C for 2 min in a water bath before adding more RNAse-free water to attain a final volume of 500 μl. An equal volume (500 μl) of solution mixture containing water-phenol, chloroform, and isopentanol in the ratio of 25:24:1 was added to the RNA suspension and mixed gently before proceeding to centrifugation at 10,000 rpm for 10 min at 4°C. About 200 μl of supernatants was pipetted into new sterilized Eppendorf tubes before adding 500 μl of absolute alcohol and stored in -80°C for 2 h. The content was then centrifuged for 10 min at 12,000 rpm at 4°C and proceeded to wash by adding 1 ml of 75% alcohol to the pellets and centrifuged at 12,000 rpm for 5 min at 4°C after which the supernatant was discarded and the precipitates were air dried under room temperature for 5 min. The air-dried precipitates are then eluted with DNA and RNA nucleotide-free water and stored in -80°C till subsequent usage for reverse transcription and for qPCR assays.

### Real-Time-PCR

The expression level of *MoKDCDH* and *MoP5CDH* in the respective knock-down strains was monitored using quantitative real-time PCR (qRT-PCR). The reverse transcription of RNAs extracted from the respective mutants from *MoKDCDH*, *MoP5CDH* knock-down lines, and the wild-type strain was carried out with SYBR^®^ Premix Ex. Taq^TM^ (Tli RNaseH Plus) purchase (Takara Biomedical Technology, Beijing Co., Ltd.). A 25 μl reaction mix was formulated as follows: 12.5 μl Premix Ex-Taq, 1 μl of each 10 μM forward and reverse primers (**Table 2**), and 1 μl cDNA template. qRT-PCR data were generated by Eppendorf Realplex2 MasterCycler (Eppendorf AG 223341, Hamburg). Data analysis was conducted using delta delta-CT (2^-ΔΔ^*^C^*^t^) method as described by Livak and Schmittgen (2001) and Zhong et al. (2015) using the expression level of actin as a positive control. The error bars represent mean ± SD, while single and double asterisks “^∗^” represent a significant reduction in fold expression of *MoKDCDH* and *MoP5CDH* in their respective knock-down strains. Consistent values were obtained with five independent biological replications and three technical replicates for each independent experiment.

**Table 2:** Primer pairs used in RT-PCR.

Gene	Primer sequence
*MoKDCDH*	F: 5′ CGTTCGTTGATTGCTTGA 3′
	R: 5′ ATGTGTGGTTGCGTTTAC 3′
*MoP5CDH*	F: 5′ GGTCTAAATGAGGGCAAAC 3′
	R: 5′ GCGTAGGTAACTGGTGAA 3′
*MoTUBULIN3*	F: 5′ TCTGACTTCAGGAATGGTC 3′
	R: 5′ AGCGGTCTGGATGTTGTTGG 3′

### Pathogenicity Assay

For plant infection assays, conidia were collected from a 7-day-old rice-bran medium. Conidial suspensions were adjusted to 1.5–2.0 × 105 conidia/ml in 0.02% Tween solution and sprayed onto 3- to 4-week-old susceptible rice seedlings (*Oryza sativa* cv. CO39). Inoculated plants were incubated in a dark, humid chamber at 25°C for 24 h before being transferred into another humid chamber with 12-h photoperiod. The plants were examined for disease symptoms after 7 days of post-inoculation (dpi). Consistent results were obtained with five independent biological experiments with three replications.

### Appressorium Formation

Appressorium formation bioassay was conducted by dropping aliquots of 20 μl conidia suspension (105) on fisher scientific hydrophobic microscope cover glass and incubated under a humid condition at a temperature of 26°C without light. Appressorium development was monitored at every 4-h interval (4, 8, 12, and 24 h) with the aid of an optical microscope or a confocal microscope; at least, 150 conidia per strain/experiment was examined; and abnormalities observed were counted. Both statistical and nonstatistical experimental data were generated from three independent biological experiments with three replicates each time with consistent results.

## Author Contributions

ZW, JN, WA, SA, and MS conceived the work, designed the experiments, and wrote the manuscript. WA, SA, LL, XC, FO, TY, and YL conducted the phenotype analysis and checked the off-target effect and the microscope examination.

## Conflict of Interest Statement

The authors declare that the research was conducted in the absence of any commercial or financial relationships that could be construed as a potential conflict of interest.

## Footnotes

1 http://pfam.xfam.org/search/sequence

2 http://smart.embl-heidelberg.de

3 https://www.genenames.org/cgi-bin/search

4 http://www.aldh.org/website/aldh/superfamily.php

5 https://www.arabidopsis.org/

6 https://shigen.nig.ac.jp/rice/oryzabase/

7 http://www.broadinstitute.org/

8 http://fungidb.org/fungidb/

9 http://smart.embl-heidelberg.de/
