# Supplementary material for: Family-Four Aldehyde Dehydrogenases Play an Indispensable Role in the Pathogenesis of Magnaporthe oryzae
Source: Front Plant Sci. 2018 Aug 8;9:980. doi: 10.3389/fpls.2018.00980 (PMC6092734; doi:10.3389/fpls.2018.00980)
Supplement: Supplementary file 1 [file Image_1.pdf]

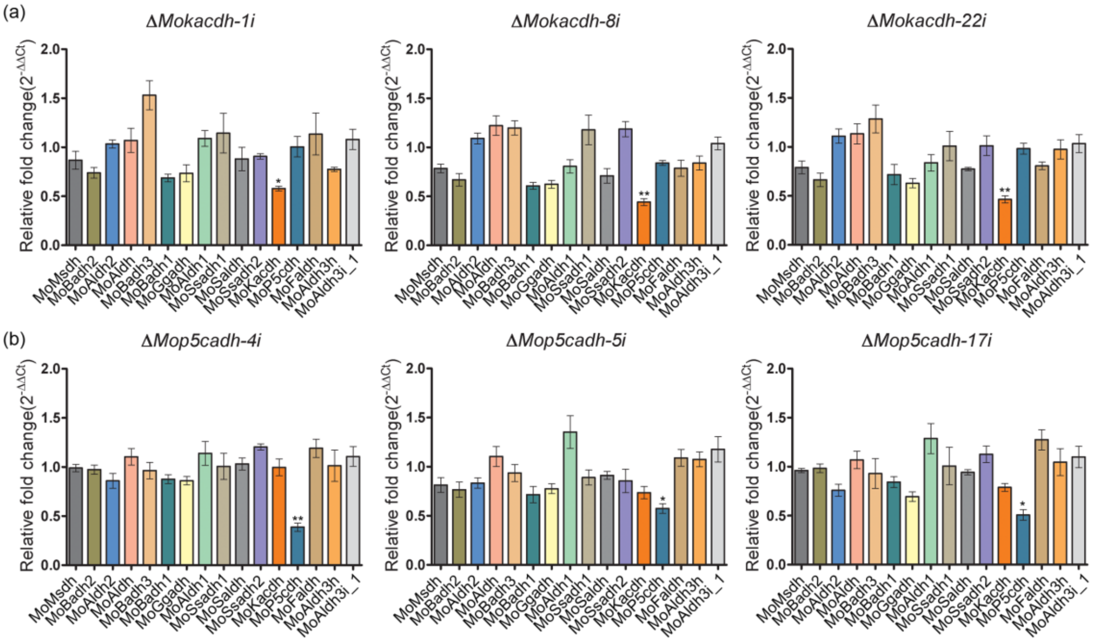

Supplementary Figure 1: **RNAi mediated silencing of MoKdcdh and MoP5cdh exert no significant influence on the expression pattern of other Aldhs:** （a）portray expression activities of 15 *M. oryzae* specific Aldhs in individual *MoKDCDH* knock-down strains (b) Represent the expression activities of 15 *M. oryzae* specific Aldhs in individual *MoP5CDH* knock-down strains. Consistent values were obtained with three independent biological experiments with three technical replicates for each independent qRT-PCR experiment. Note: The expression activities of respective Aldhs in the wild-type (Guy11) strain was used as the reference expression level in our analysis. One-way ANOVA (nonparametric) statistical analysis was carried out with graphpad prism6 and Microsoft Excel spread sheet, and the error bars represent the standard deviation. Expression level in our analysis and single "*" and double "**" asterisks represent significant differences existing between Guy11 and the respective knock-down mutants ($P < 0.05$) and ($P < 0.01$).
